# Supplementary material for: Prevalence and risk factors of Helicobacter pylori infection among children in Kuichong Subdistrict of Shenzhen City, China
Source: PeerJ. 2020 Apr 3;8:e8878. doi: 10.7717/peerj.8878 (PMC7134012; doi:10.7717/peerj.8878)
Supplement: Supplemental Information 1 [file peerj-08-8878-s001.docx]

**儿童生活习惯调查问卷（家长用）**

【填写须知】

本研究调查符合深圳大学附属深圳市第二人民医院道德伦理审查和批准程序。任何参与该项调查的人员都可以随时向工作人员提出疑问。任何对工作人员的反应不满意的参与者以提起投诉。

1．选择题：请将您在认为正确的选项上打“√”

2．填空题：请直接在横线上添写选项或文字，没有的可填“0”。

1. 个人基本资料

儿童姓名 性别 年龄 家长电话

就读学校 儿童身份证号 民族

体重 kg身高 cm 头围 cm 胸围 cm

最近一次文化课考试成绩等级 □ A □ B □ C □ D

上一年度体质健康等级评定 □优秀 □良好 □合格 □不合格

儿童出生地 □深圳

□非深圳，请填写具体出生地 来深圳生活的年龄 岁

父亲出生地 母亲出生地 （省、市、乡）

家庭孩子数量 □1人 □2-3人 □>3人

家庭人口数 □≦3人 □4-5人 □≧6人

父亲文化程度 □高中、中专及以下 □专科及本科 □硕士及以上

母亲文化程度 □高中、中专及以下 □专科及本科 □硕士及以上

家庭总收入（元/年） □<10万 □10-20万 □>20万

人**均**居住面积 （平方米） □ <20/人 □ 20-40/人 □ >40/人

1. 儿童及家庭成员生活习惯

1.该儿童0-3岁是谁在喂养 □母亲 □父亲 □祖/外祖父母 □保姆

2.该儿童刷牙习惯 □0-3次/周 □4-6次/周 □7次/周及以上

3.该儿童有无龋齿 □无 □有 （龋齿数量）

5.该儿童既往有无HP感染 □无 □有 □不清楚

6.母亲有无HP感染 □无 □有 □不清楚

7.父亲有无HP感染 □无 □有 □不清楚

8.家庭其他成员有无HP感染 □无 □有

9.家庭成员中有无烧心或胃痛、反酸症状 □无 □有

10.胃癌家族史 □无 □有

11.孩子出生方式 □自然分娩 □剖腹产

12.母乳喂养持续时间 □无 □<3个月 □3-6个月 □≥6个月

13.使用奶瓶是如何测试温度的

□腕部/手臂测试 □嘴部测试 □温度计

14.婴幼儿期辅食喂养方式 □喂养者咀嚼后喂养 □与喂养者共用餐具

（可多选） □儿童专用餐具

15.儿童吸吮手指习惯 □无 □有

16.外出就餐习惯(中晚餐) □0-1次/周 □2-4次/周 □5次/周及以上

17.餐前便后洗手 □0-1次/天 □2-4次/天 □5次/天及以上

18.是否食生冷果蔬**是否**削皮 □否 □是，基本都削皮

19.是否经常饮用**未**经过烧开的水 □否 □是，经常喝**没**烧开的水

20.家人与孩子共用餐具习惯 □否 □是，共用餐具，无公筷

21.家人与孩子共用刷牙口杯 □否 □是

22.家中餐具消毒柜消毒 □否 □是

23.家中养宠物 □无 □有 （请说明具体动物）

24.家里饮水来源 □自来水 □瓶装水 □井水 □河水或湖水

25.家中是否安装净水设备 □无 □有安装

26.儿童喜食零食 □0-1次/周 □2-4次/周 □5次/周及以上

27.儿童进食水果频率 □0-1次/周 □2-4次/周 □5次/周及以上

三、儿童的不适症状

腹胀 □0-1次/周 □2-4次/周 □5次/周及以上

腹痛 □0-1次/周 □2-4次/周 □5次/周及以上

打饱嗝 □0-1次/天 □2-4次/天 □5次/天及以上

腹泻 □0-1次/周 □2-4次/周 □5次/周及以上

口臭 □无 □有

贫血 □无 □有

皮肤过敏□无 □有

哮喘 □无 □有

其他 （如孩子有其他不适症状，请具体说明）
