# Supplementary material for: Prevalence and risk factors of Helicobacter pylori infection among children in Kuichong Subdistrict of Shenzhen City, China
Source: PeerJ. 2020 Apr 3;8:e8878. doi: 10.7717/peerj.8878 (PMC7134012; doi:10.7717/peerj.8878)
Supplement: Supplemental Information 3 [file peerj-08-8878-s003.docx]

**Table S2:** List of sociodemographic, lifestyle and clinical variables that had no significant influence on *H. pylori* infection status in 1355 children.

| Variables | *H. pylori*  positive (%) | *H. pylori*  negative (%) | *p* |
| --- | --- | --- | --- |
| Feeder |  |  | 0.981 |
| Parents | 172 (16.7) | 855 (83.3) |  |
| Grandparents | 36 (16.1) | 187 (83.9) |  |
| Both parents and grandparents | 16 (17.6) | 75 (82.4) |  |
| Others | 2 (20) | 8 (80) |  |
|  |  |  |  |
| Family income per annum (RMB) |  |  |  |
| Up to 200,000 | 208 (17.1) | 1007 (82.9) | 0.2 |
| >200,000 | 18 (12.9) | 122 (87.1) |  |
|  |  |  |  |
| Habit of sucking fingers |  |  | 0.503 |
| No | 159 (16.3) | 819 (83.7) |  |
| Yes | 67 (17.8) | 310 (82.2) |  |
|  |  |  |  |
| Pet |  |  | 0.105 |
| No | 207 (16.3) | 1064 (83.7) |  |
| Yes | 19 (23.2) | 63 (76.8) |  |
|  |  |  |  |
| Drinking unboiled water |  |  | 0.933 |
| No | 203 (16.7) | 1012 (83.3) |  |
| Yes | 23 (16.4) | 117 (83.6) |  |
|  |  |  |  |
| Frequency of brushing teeth |  |  | 0.698 |
| 0-3 times per week | 24 (18.2) | 108 (81.8) |  |
| 4-6 times per week | 37 (15) | 209 (85) |  |
| ＞7 times per week | 165 (16.9) | 812 (83.1) |  |
|  |  |  |  |
| Washing hands before eating and after going to washroom |  |  | 0.552 |
| Rarely | 17 (15.9) | 90 (84.1) |  |
| Sometimes | 101 (15.7) | 544 (84.3) |  |
| Always | 108 (17.9) | 495 (82.1) |  |
|  |  |  |  |
| Frequency of eating fruits |  |  | 0.884 |
| 0-1 time per week | 19 (15.7) | 102 (84.3) |  |
| 2-4 times per week | 126 (17.1) | 610 (82.9) |  |
| ＞5 times per week | 81 (16.3) | 417 (83.7) |  |
|  |  |  |  |
| Abdominal bloating |  |  | 0.902 |
| 0-1 time per week | 219 (16.7) | 1096 (83.3) |  |
| 2-4 times per week | 5 (16.1) | 26 (83.9) |  |
| >=5 times per week | 2 (22.2) | 7 (77.8) |  |
|  |  |  |  |
| Abdominal pain |  |  | 0.911 |
| 0-1 time per week | 215 (16.6) | 1081 (83.4) |  |
| 2-4 times per week | 9 (18.4) | 40 (81.6) |  |
| >=5 times per week | 2 (20) | 8 (80) |  |
|  |  |  |  |
| Diarrhea |  |  | 0.5 |
| 0-1 time per week | 223 (16.7) | 1114 (83.3) |  |
| 2-4 times per week | 3 (23.1) | 10 (76.9) |  |
| >=5 times per week | 0 (0) | 5 (100) |  |
|  |  |  |  |
| Burping |  |  | 0.507 |
| 0-1 time per week | 208 (16.5) | 1052 (83.5) |  |
| 2-4 times per week | 16 (20.8) | 61 (79.2) |  |
| >=5 times per week | 2 (11.1) | 16 (88.9) |  |
|  |  |  |  |
| Anemia |  |  | 0.689 |
| No | 204 (16.8) | 1009 (83.2) |  |
| Yes | 22 (15.5) | 120 (84.5) |  |
|  |  |  |  |
| Skin allergies |  |  | 0.83 |
| No | 206 (16.6) | 1034 (83.4) |  |
| Yes | 20 (17.4) | 95 (82.6) |  |
|  |  |  |  |
| Asthma |  |  | 0.777 |
| No | 224 (16.7) | 1121 (83.3) |  |
| Yes | 2 (20) | 8 (80) |  |
|  |  |  |  |
| Duration of breastfeeding |  |  | 0.523 |
| Nil | 26 (15.1) | 146 (84.9) |  |
| Up to 6 months | 83 (18.2) | 372 (81.8) |  |
| More than 6 months | 117 (16.1) | 611 (83.9) |  |
|  |  |  |  |
| Delivery mode |  |  | 0.376 |
| Natural birth | 146 (17.4) | 694 (82.6) |  |
| Caesarean birth | 80 (15.5) | 435 (84.5) |  |
